# Serotonin Syndrome Triggered by Increasing the Dose of Quetiapine

**DOI:** 10.5811/cpcem.2021.5.52505

**Published:** 2021-08-05

**Authors:** Yayoi Miyamatsu, Ryutaro Tanizaki

**Affiliations:** * Nabari City Hospital, Department of Internal Medicine, Nabari, Japan; † Mie University School of Medicine, Department of Community Medicine, Nabari, Japan

**Keywords:** serotonin syndrome, quetiapine, tremor, neuroleptic malignant syndrome

## Abstract

**Case presentation:**

An 85-year-old woman with a history of depression treated with polypharmacy including selective serotonin reuptake inhibitor presented to the emergency department with head, and upper and lower limb tremors four hours after increasing the dose of quetiapine from 12.5 milligrams (mg) per day to 25 mg/day. She was diagnosed with serotonin syndrome (SS), and all medications except clotiazepam were discontinued. The symptoms subsided within 48 hours.

**Discussion:**

The use of atypical antipsychotics alone seldom increases the risk of SS. However, combining atypical antipsychotics with serotonergic agents increases the risk of SS because the activity of serotonin receptor subtype 1A is relatively enhanced. This report suggests that physicians should be aware that even a small increase in quetiapine could pose a risk of developing SS.

## CASE PRESENTATION

An 85-year-old woman presented to the emergency department with acute head and upper and lower limb tremors and agitation. She had a long history of major depressive disorder and insomnia, which were treated with escitalopram 20 milligrams (mg) per day, mirtazapine 30 mg/day, sulpiride 20 mg/day, olanzapine 2.5 mg/day, quetiapine 12.5 mg/day, and clotiazepam 5 mg/day. Her symptoms occurred four hours after the dose of quetiapine was increased from 12.5 to 25 mg/day by her psychiatrist to improve her insomnia. All doses had remained unchanged except for that of quetiapine. Physical examination revealed fever of 39.1℃, tachycardia, agitation, mydriasis, deep tendon hyperreflexia, and symmetrical tremor of the head, and upper and lower limbs that lasted for 15 seconds and repeated at five-second intervals (video).

There was no muscular rigidity. Laboratory findings showed normal white blood cells, C-reactive protein, and creatinine phosphokinase levels. A computed tomography of the head was performed and did not reveal any significant abnormalities. Serotonin syndrome (SS) was then diagnosed based on the Hunter Seratonin Toxicity Criteria,^1^ and she was admitted to our hospital. All medications except clotiazepam were discontinued, and the symptoms subsided within 48 hours.

## DISCUSSION

Serotonin syndrome is related to overstimulation of a serotonin receptor subtype 1A (5-HT_1A_), commonly caused by the use of serotonergic agents.^1^ Quetiapine is an atypical antipsychotic agent that exhibits serotonergic receptor 5-HT_2A_ antagonism, which has a short half-life and is often used to manage agitation and psychotic symptoms in hyperactive delirium.^2^ The use of atypical antipsychotics alone seldom increases the risk of SS; specifically, there is a lower risk of SS on treatment with quetiapine because it is a significantly weaker 5-HT_2A_ antagonist than other atypical antipsychotics.^3^ However, combining atypical antipsychotics and serotonergic agents increases the risk of SS because the activity of 5-HT_1A_ is relatively enhanced.

Furthermore, because quetiapine also exhibits a dopaminergic D2 receptor antagonism, which may cause neuroleptic malignant syndrome (NMS),^4^ it was necessary to differentiate between SS and NMS in this patient. In general, agitation, diarrhea, mydriasis, myoclonus, and hyperreflexia are more frequent in SS, whereas dysphagia, hypersalivation, incontinence, hyperthermia, akinesia, lead pipe rigidity, and rhabdomyolysis are characteristics of NMS.^5^ Thus, it is not challenging to differentiate between SS and NMS in a patient with typical symptoms. However, it should be noted that atypical NMS, which lacks typical NMS symptoms, cannot be excluded based on the symptoms alone. The significant difference between SS and NMS is time to onset. Neuroleptic malignant syndrome generally appears within seven days following the introduction of a neuroleptic agent, whereas SS develops rapidly within 24 hours following the introduction of a serotonergic agent.^5^ In this patient, the diagnosis of SS was made because of rapid onset four hours after increasing the dose of quetiapine, in addition to her symptoms being consistent with the Hunter criteria.^1^

Management of SS mainly involves supportive care, the discontinuation of serotonergic drugs, and treatment with benzodiazepines. Immediate sedation, neuromuscular paralysis, and orotracheal intubation are performed in severe cases.^3^ In this patient, all medications except clotiazepam were discontinued, and the symptoms resolved within 48 hours. Although quetiapine has a lower risk of SS than other atypical antipsychotics, physicians should be aware that even a small increase in quetiapine could pose a risk of developing SS.

CPC-EM Capsule *Pending*

What do we already know about this clinical entity?

*Serotonin syndrome is commonly caused by serotonergic agents*.

What is the major impact of the image(s)?

*Even a small increase in quetiapine could cause serotonin syndrome in patients taking serotonergic agents*.

How might this improve emergency medicine practice?

*Physicians in the emergency department can recognize that even a small increase in quetiapine could pose a risk of developing serotonin syndrome*.

## Supplementary Information

Video An agitated patient, repeatedly calling the unidentified name “Etsuko.” and demonstrating intermittent tremor of her head, and upper and lower limbs after escalation of her quetiapine dose
